# Evaluation of Toxic, Cytotoxic, Mutagenic, and Antimutagenic Activities of Natural and Technical Cashew Nut Shell Liquids Using the *Allium cepa* and *Artemia salina* Bioassays

**DOI:** 10.1155/2015/626835

**Published:** 2015-03-10

**Authors:** Aracelli de Sousa Leite, Alisson Ferreira Dantas, George Laylson da Silva Oliveira, Antonio L. Gomes Júnior, Sidney Gonçalo de Lima, Antônia Maria das Graças Lopes Citó, Rivelilson M. de Freitas, Ana Amélia de C. Melo-Cavalcante, José Arimateia Dantas Lopes

**Affiliations:** ^1^Laboratório de Pesquisa em Genética Toxicológica de Pós-Graduação em Ciências Farmacêuticas da Universidade Federal do Piauí, 6409-550 Teresina, PI, Brazil; ^2^Programa de Pós-Graduação em Biotecnologia (RENORBIO) da Universidade Federal do Piauí, 6409-550 Teresina, PI, Brazil; ^3^Programa de Pós-Graduação em Biologia Animal, Departamento de Genética e Morfologia, Instituto de Ciências Biológicas, Universidade de Brasília, 70910-900 Brasília, DF, Brazil; ^4^Laboratório de Pesquisa em Neuroquímica Experimental do Programa de Pós-Graduação em Ciências Farmacêuticas da Universidade Federal do Piauí, 6409-550 Teresina, PI, Brazil; ^5^Departamento de Química, CCN, Universidade Federal do Piauí, 6409-550 Teresina, PI, Brazil

## Abstract

The cashew nut releases a substance that is known as cashew nut shell liquid (CNSL). There are both natural (iCNSL) and technical (tCNSL) cashew nut shell liquids. This study used an *Artemia salina* bioassay to evaluate the toxic effects of iCNSL and tCNSL cashew nut shell liquids. It also evaluated the toxicity, cytotoxicity, and mutagenicity of CNSL and its effects on the damage induced by copper sulfate (CuSO_4_·5H_2_O) on the meristems' root of *Allium cepa*. Effects of the damage induced by CuSO_4_·5H_2_O were evaluated before (pre-), during (co-), and after (post-) treatments. The iCNSL contained 94.5% anacardic acid, and the tCNSL contained 91.3% cardanol. The liquids were toxic to *A. salina*. Toxicity, cytotoxicity, and mutagenicity were observed with iCNSL compared with the negative control. Similarly, iCNSL failed to inhibit the toxicity and cytotoxicity of CuSO_4_·5H_2_O. The tCNSL was not toxic, cytotoxic, or mutagenic in any of the concentrations. However, the lowest iCNSL concentrations and all of the tCNSL concentrations had preventive, antimutagenic, and reparative effects on micronuclei and on chromosomal aberrations in the *A. cepa*. Therefore, protective, modulating, and reparative effects may be observed in the *A. cepa*, depending on the concentration and type of CNSL used.

## 1. Introduction

Recent epidemiological studies have shown that medicinal plants may be involved in preventing or delaying the development of various diseases [1, 2]. These plants may act on different targets in signal transduction pathways that may modulate gene expression, cell cycle progression, cellular proliferation, and/or apoptosis [3, 4]. However, adverse effects, such as genotoxicity, mutagenicity, and carcinogenicity [5, 6], can also occur. These effects may be triggered by compounds that interact with deoxyribonucleic acid (DNA), which would cause cellular toxicity and/or genotoxicity [7].

The species* Anacardium occidentale *(Anacardiaceae) is found in tropical regions worldwide. It is common in Brazil, India, Mozambique, Tanzania, Kenya, Vietnam, Indonesia, and Thailand [8]. Recent studies have been associated with several biological effects of the cashew plant. It can be used as an antioxidant [9, 10] and can be used in dermatitis [11] and also possesses larvicidal [12], antigenotoxic [13], and antimicrobial [14] activities.

The cashew nut releases a liquid that is known as cashew nut shell liquid (CNSL). This liquid is a natural source of phenolic compounds that contribute to its antioxidant [15, 16], antifungal [17], antibacterial [18], larvicidal [19], and nongenotoxic effects in prokaryotic [20, 21] and eukaryotic cells [22]. This liquid is classified into two categories, depending on the extraction method used: natural CNSL (iCNSL) extracted with solvents, and its main components are anacardic acid (62.9%), cardol (23.98%), and cardanol (6.99%) [23] and technical CNSL (tCNSL) wich is prepared by burning the nuts industrially at high temperatures and it contains cardanol (60–65%), cardol (15–20%), polymeric material (10%), and small amounts of metilcardol [24].

The present study aimed to evaluate the toxicity of iCNSL and tCNSL to* A. salina* and to determine their toxic, cytotoxic, and mutagenic actions and their protective effects against the damage that is induced by CuSO_4_·5H_2_O in* A. cepa* root meristems.

## 2. Material and Methods

### 2.1. CNSL Preparation and Doses Choice

Ripe cashew nuts were collected from cashew trees in Teresina in the state of Piauí, Brazil. For iCNSL, extraction the ripe cashew nuts were stored in styrofoam with liquid nitrogen for 5 minutes. Then, they were crushed and subjected to hot extraction Soxhlet extractor with hexane for 16 hours. The iCNSL was then concentrated in a rotary evaporator at 45°C. The tCNSL was provided by a company of the Group Europa–Castanha located in Altos, Teresina, Piauí, in northeastern Brazil. According to the company, the nuts were immersed in a hot bath at 195°C for 3 hours to extract the tCNSL. The tCNSL was then filtered and stored.

The lowest doses used in this study were chosen because recent research on the tCNSL (100–500 *µ*g/mL) has shown antioxidant properties. Thus, doses lower than those tested in the literature have been evaluated to check if they would still be antioxidant and nonmutagenic.

### 2.2. CNSL Methylation Reaction

Samples of the extracted iCNSL and tCNSL were analysed by gas chromatography coupled to mass spectrometry (GC-MS) in the form of methyl esters. Five milligrams of CNSL was dissolved in 0.5 mL of diethyl ether and transferred to a 5.0 mL flask. A solution of diazomethane in ether (2.0 mL) was then added dropwise at low temperature in an ice bath until outgassing was no longer observed. The flask was sealed with a ground glass stopper and magnetically stirred continuously at room temperature. After 3 hours, the reaction was monitored by thin layer chromatography (TLC). Following ether evaporation, the residue was solubilised in ethyl acetate and analysed by GC-MS [25].

### 2.3. GC-MS Analysis

Both of the derived CNSL samples were then analysed in a GC-MS system (Shimadzu, GC-17A/MS-QP5050A). The column chromatography DB-5HT (J & W Scientific) was 30 m long and 0.25 mm in diameter, had a film thickness of 0.10 *µ*m, and used helium as a carrier gas (1.0 mL/min). The following parameters were used: interface = 270°C, injector = 250°C, initial temperature = 60°C (2 min), 4.0 mL/min to 180°C (4 min), and 10 mL/min to 260°C (10 min). Identification was achieved by comparing mass spectra (43 to 4500 Daltons, electron impact ionisation, 70 eV) and data from the literature of De Lima et al. [25].

### 2.4. *Artemia salina* Test

The method used to assess the toxicity of iCNSL and tCNSL on* A. salina* was adapted from Meyer et al. [26]. The microcrustaceans were hatched in water as mentioned in Materials and Methods for 48 hours until their larvae were released. Ten* A. salina* specimens were introduced into each of the three tubes containing a 17.37, 34.75, or 69.50 *μ*g·mL^−1^ concentration of iCNSL or tCNSL. A nonactive substance (1 : 1 seawater and mineral water) was used as a control. The dead specimens were counted under a stereomicroscope after 24 hours.

### 2.5. *Allium cepa* Test

The* A. cepa* test was adapted from the method reported by Fiskesjö [27]. Each experimental group consisted of iCNSL and tCNSL at concentrations of 17.37, 34.75, or 69.50 *μ*g·mL^−1^, as well as a solution of 500 *µ*g/mL Tween 20 (solvent), a negative control (dechlorinated water), and a positive control (1.2 *μ*g·mL^−1^ copper sulphate). Small bulbs of* A. cepa* (2*n* = 16) were purchased from supermarkets in Teresina, Piauí.

Following 72 hours of exposure, the roots were measured in centimetres to assess toxicity. The roots were then placed in Carnoy's fixative solution (ethanol/glacial acetic acid 3 : 1 v/v), refrigerated at 4°C for 24 hours, followed by 70% ethanol solution and refrigeration. The roots were subsequently hydrolysed in a hydrochloric acid solution (1 N) and placed in a staining solution (Schiff's dye) for two hours.

The roots were then placed on slides and sectioned in the meristem region. This region of the root was stained with 2% acetic carmine, covered with a cover slip, and then observed under an optical microscope (1000x) to analyse cytotoxicity, mutagenicity, and the effects on the damage induced by copper sulphate. A total of 1,000 cells were analysed on each slide. The following parameters were observed: (a) mitotic index (MI), (b) the frequency of chromosomal aberrations (CA) in anaphase and telophase, and (c) the frequency of micronuclei (MN).

### 2.6. Effects on Copper Sulphate-Induced Damage

The method used to evaluate antimutagenicity by means of the* A. cepa* test was adapted from Malini et al. [28]. The present study used CuSO_4_·5H_2_O as the genotoxic agent because of its mutagenic potential [29, 30].

Three types of treatments were standardised: (1) pretreatment, in which the bulbs were exposed initially to iCNSL or tCNSL for 48 hours and the roots were then washed in distilled water and placed in 1.2 *μ*g·mL^−1^ CuSO_4_·5H_2_O solutions for 24 hours to germinate; (2) cotreatment, in which the bulbs were placed in tubes that contained iCNSL or tCNSL and a 1.2 *μ*g·mL^−1^ CuSO_4_·5H_2_O solution at a 1 : 1 ratio for 72 hours for germination; and (3) posttreatment, in which the bulbs were first placed in 1.2 *μ*g·mL^−1^ CuSO_4_·5H_2_O solution for 48 hours, and then the roots were rinsed with distilled water and placed in tubes that contained iCNSL or tCNS for 24 hours for germination. The samples were then processed in the similar way as mentioned in the* A. cepa* test.

### 2.7. Statistical Analysis

The number of dead* A. salina* was analysed using the Statistical Package for the Social Sciences (SPSS) software, version 17:0. The IC_50_ was assessed by probit analysis. The tests were performed in triplicate. The data were analysed using GraphPad Prism software (version 6.03), and the experimental groups were compared with the negative and positive control groups. All results were expressed as mean ± standard deviation (SD). The data were assessed by an analysis of variance (ANOVA) followed by Tukey's test for multiple comparisons for genotoxicity and mutagenicity tests The significance levels were ^*^ *P* < 0.05, ^**^ *P* < 0.01, and ^***^ *P* < 0.001.

## 3. Results

### 3.1. CNSL Chromatographic Analyses

**Figure 1:**
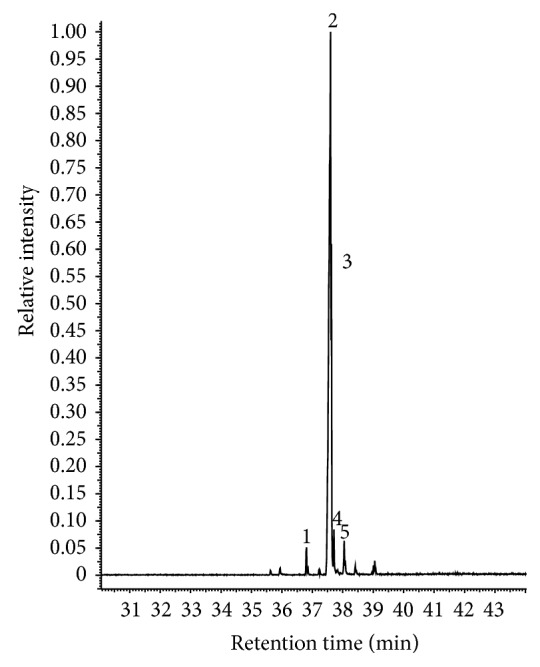
The chromatographic profile of iCNSL obtained by GC-MS analysis. 1: metilcardol, 2: monounsaturated anacardic acid, 3: diunsaturated anacardic acid, 4: anacardic acid, and 5: unidentified.

**Figure 2:**
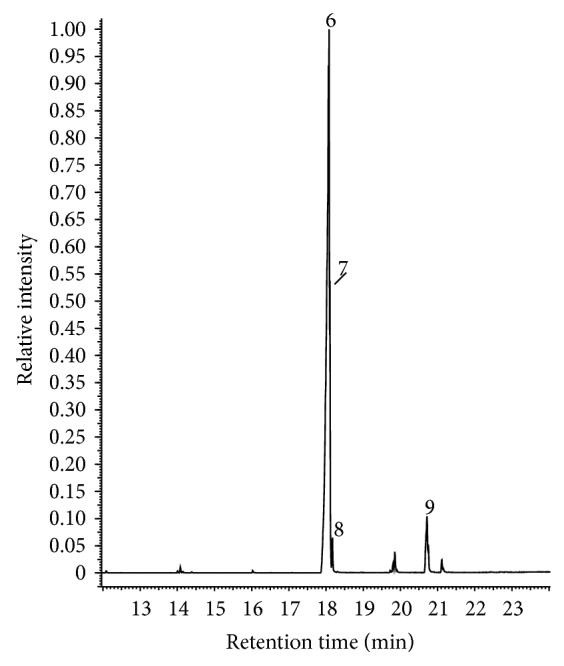
The chromatographic profile of tCNSL obtained by GC-MS analysis. 6: monounsaturated cardanol, 7: diunsaturated cardanol, 8: cardanol, and 9: unidentified.

The main phenolic compounds present in both types of CNSL were identified by GC-MS analysis. The iCNSL contained metilcardol (2.90%), monounsaturated anacardic acid (82.90%), diunsaturated anacardic acid (8.0%), anacardic acid (3.60%), and 2.60% unidentified compounds (Table 1 and Figure 1). The tCNSL exhibited 79.40% monounsaturated cardanol, 8.67% diunsaturated cardanol, 3.23% cardanol, and 8.70% unidentified compounds (Table 1 and Figure 2). These results indicate that the main phenolic compound of iCNSL is monounsaturated anacardic acid and monounsaturated cardanol for the tCNSL.

### 3.2. Evaluation of iCNSL and tCNSL Toxicity on* A. salina*

**Figure 3:**
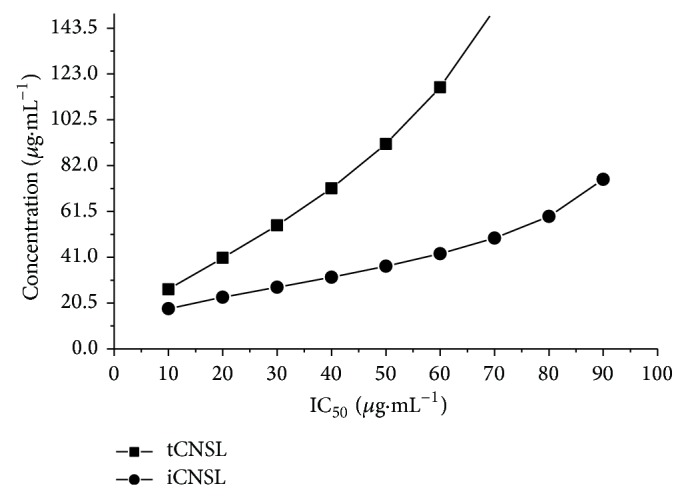
The lethal concentration (LC_50_) of iCNSL and tCNSL in* A. salina* toxicity test. Mean of three independent experiments at different iCNSL and tCNSL concentrations.

The toxicity of iCNSL and tCNSL on* A. salina* was evaluated, and the 50% lethal concentration (IC_50_) values are shown in Figure 3. The IC_50_ of iCNSL and tCNSL was 36.96 *μ*g·mL^−1^ and 91.67 *μ*g·mL^−1^, respectively.

### 3.3. Evaluation of iCNSL and tCNSL Toxicity and Cytotoxicity in* A. cepa*

**Figure 4:**
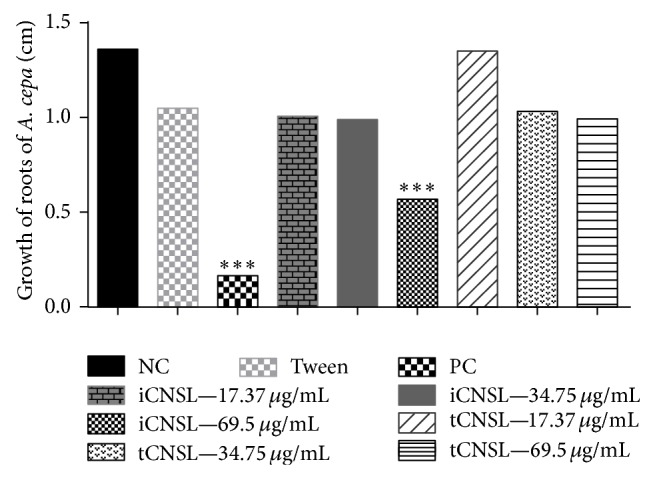
Toxic effects of iCNSL and tCNSL observed by roots growth in* A. cepa* (cm). Significant compared with the negative control. ^*^ *P* < 0.05; ^**^ *P* < 0.01 ANOVA. Tukey's test for multiple comparisons between groups.

Analysis of the macroscopic parameter (root growth), which is affected by toxicity in* A. cepa* root meristems, showed that iCNSL had a significant toxic effect at the highest test concentration (69.50 *μ*g·mL^−1^) compared with the negative control (*P* < 0.001). However, tCNSL had no toxic effect (*P* > 0.05) (Figure 4).

When* A. cepa* root meristems were initially exposed to iCNSL or tCNSL in pretreatment and were then treated with a CuSO_4_·5H_2_O solution at a 1.2 *μ*g·mL^−1^ concentration, iCNSL failed to prevent the toxicity of the CuSO_4_·5H_2_O and tCNSL (cotreatment) protected against copper sulphate toxicity only at the highest concentration (69.50 *μ*g·mL^−1^). However, both CNSLs (iCNSL and tCNSL) showed co- or posttreatment effects on CuSO_4_·5H_2_O toxicity (Table 2).

**Figure 5:**
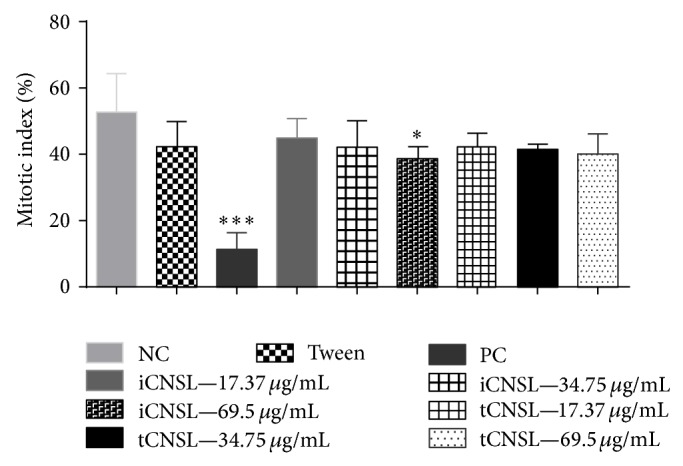
Evaluation of cytotoxic effects of iCNSL and tCNSL observed by inhibition of mitotic index in roots of* A. cepa*. Significant compared with the negative control. ^*^ *P* < 0.05; ^**^ *P* < 0.01 ANOVA. Tukey's test for multiple comparisons between groups.

The mitotic indices (MIs) of* A. cepa* root meristems that were exposed to different iCNSL and tCNSL concentrations are shown in Figure 5. The iCNSL significantly inhibited (*P* < 0.05) cell division in* A. cepa* root meristems at only the highest test concentration (69.50 *μ*g·mL^−1^). The tCNSL had no cytotoxic effects on MI at any of the test concentrations.

Both iCNSL and tCNSL failed to prevent copper sulphate cytotoxicity in* A. cepa *root meristems in pretreatment, and neither modulated the cytotoxicity of copper sulphate in cotreatment (Table 3).

Although iCNSL and tCNSL inhibited (*P* < 0.001) the cytotoxic activity of copper sulphate at the highest posttreatment test concentration, they also significantly reduced* A. cepa* root growth (*P* < 0.001) at this higher concentration (Table 3). These data indicate that neither iCNSL nor tCNSL showed preventive, modulating, and reparative activity against the cytotoxicity induced in* A. cepa* root meristems by CuSO_4_·5H_2_O.

### 3.4. Evaluation of iCNSL and tCNSL Mutagenicity and Their Effects on Copper Sulphate

Only iCNSL at the highest test concentration (69.50 *µ*g·mL^−1^) induced MN mutagenicity in* A. cepa* root meristems (*P* < 0.05) compared with the negative control. The two lower concentrations of iCNSL and all three concentrations of tCNSL failed to induce MN mutagenicity in* A. cepa* root meristems (*P* > 0.05) (Table 4).

Both iCNSL and tCNSL protect (pretreatment) the DNA of* A. cepa* root meristems, as observed by the inhibition of MN formation induced by CuSO_4_·5H_2_O. However, iCNSL at the highest test concentration did not confer this protection. Similarly, antimutagenic and reparative effects were evidenced by a reduction in MN in co- and posttreatments, except at the highest iCNSL concentration (Table 4). Both iCNSL and tCNSL strongly affected the prevention, modulation, and repair of damage induced by CuSO_4_·5H_2_O in* A. cepa* meristems.

**Figure 6:**
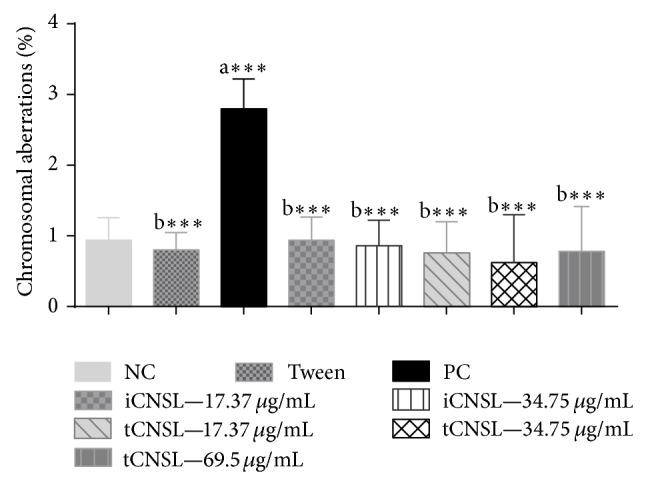
Mutagenic effects of iCNSL and tCNSL by frequency of chromosomal aberrations in* A. cepa* roots. ^a^Significant compared with the negative control; ^b^Significant compared with the positive control. ^***^ *P* < 0.001 ANOVA. Tukey's test for multiple comparisons between groups.

The mutagenicity of the highest iCNSL concentration (69.50 *µ*g·mL^−1^) was not confirmed when analysing the frequency of chromosomal aberration (CA) because iCNSL at this concentration had already been shown to have mutagenic effects as evidenced by increased MN frequency, and MI inhibition also precluded observation of CA at this concentration. Neither iCNSL nor tCNSL showed mutagenic effects as measured by the frequency of CA compared to the positive control (Figure 6).

**Figure 7:**
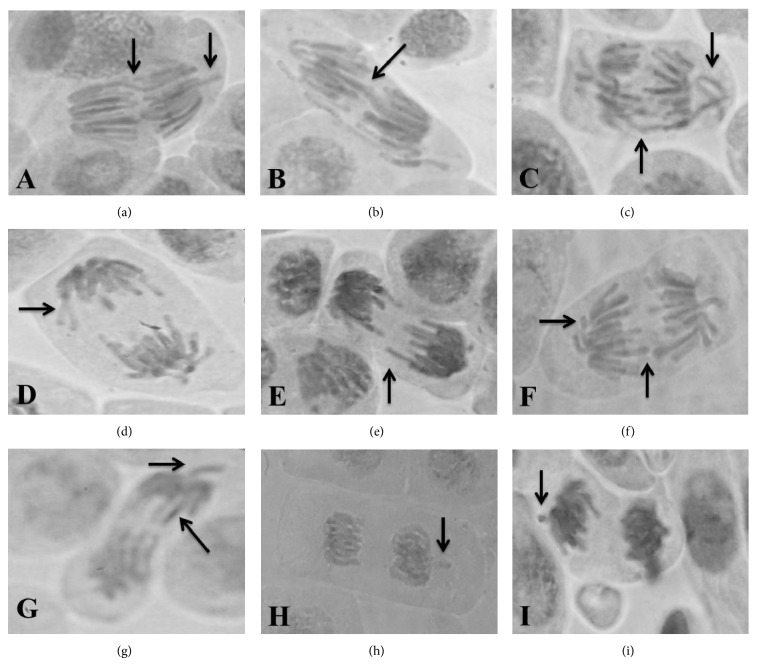
The photomicrographic profile of* A. cepa *root meristems in which pre-, co-, and posttreatment with iCNSL and tCNSL provided protection against the damage caused by exposure to copper sulphate (at a concentration of 1.2 *μ*g/mL). (a) A cell in anaphase with a chromosome bridge and a chromosome fragment, (b) a cell in anaphase with a chromosome bridge, (c) a cell in anaphase with a chromosome bridge and a vagrant chromosome, (d) and (e) cells in anaphase with laggard chromosomes, (f) a cell in anaphase with laggard chromosomes and chromosome fragments, (g) cells in anaphase with laggard and vagrant chromosomes, and (h) and (i) cells in telophase with chromosome fragments.

Chromosome bridges, vagrant and laggard chromosomes, and chromosome fragments are the most notable of the CAs induced by CuSO_4_·5H_2_O during anaphase and telophase. The photomicrographic profile of CAs identified in* A. cepa* root meristems exposed to CuSO_4_·5H_2_O is represented in Figure 7. This damage was modulated by iCNSL and tCNSL at the tested concentrations, which suggests that both may have the inhibition mechanisms of aneugenic and/or clastogenic agents.

Moreover, both types of CNSL showed protective, antimutagenic, and DNA-repair effects in regard to the damage induced by copper sulphate (Table 5).

## 4. Discussion

Genomic instability is a common cause of cancer. Cancer cells are more susceptible than normal cells to DNA-damaging agents. This increased susceptibility provides a path for therapeutic intervention. Phytochemicals may affect the genome and trigger damage to DNA and repair mechanisms [31]. However, many of such dietary substances have been associated with a decreased risk of cancer, for example, breast cancer [32, 33].

Differences between the two types of CNSL were identified by the GC-MS analysis. The iCNSL contained a mixture of anacardic acids that constituted 94.5% of its composition. Conversely, tCNSL exhibited a mixture of cardanols that constituted more than 90% of its composition (Table 1). These percentages corroborate the studies by Philip et al. [34], who reported that anacardic acid may exceed 80% of iCNSL composition. This percentage may be as high as 90%, according to Das and Ganesh [35]. Previous studies had found that cardanol is the main component of tCNSL [24, 36]. The difference in chemical composition between the two types of CNSL might result from the preparation process used to obtain tCNSL, which involves heating the cashew nuts to 180–200°C. This heat causes anacardic acid to undergo decarboxylation and conversion into cardanol, leading to the higher cardanol concentration in tCNSL [37].

The chemical compositions of both iCNSL and tCNSL recorded in the present study differ from the chemical composition reported in the literature [15, 23, 24]. The origin of the cashew nuts, the weather conditions, and, particularly, the extraction process used may account for the differences between this and other studies with regard to the ratio of phenolic compounds found in iCNSL [35], and the operating and heating conditions may affect the ratios recorded in tCNSL [38].

The* A. salina* bioassay is considered to be useful for preliminary assessments of general toxicity, and it correlates well with cytotoxic activity against some types of solid tumours in humans [26]. The IC_50_ of iCNSL in* A. salina* was lower than that of tCNSL (Figure 3). Plant extracts with LC_50_ values under 1,000 *μ*g·mL^−1^ are considered to be active and to have toxic activity [39, 40]. Therefore, although iCNSL and tCNSL had different LC_50_ values, both are considered to have shown toxic effects in the acute toxicity test with* A. salina*. Guerra [41] suggests that the* Artemia* bioassay could be used for the toxicity evaluations of compounds that are rich in phenols.

The most significant toxicity results found in this study (Figure 3) were obtained when* A. salina* was treated with iCNSL. This finding may be related to the presence of anacardic acids in iCNSL because anacardic acids have been reported to be cytotoxic [22]. Other studies have reported that anacardic acid derivatives, such as isonicotinoyl hydrazone, show* in vitro *activity against* Mycobacterium smegmatis* [42].

Muroi et al. [43] showed that different types of unsaturations present in the chain of anacardic acids are related to increased antibacterial activity against* Staphylococcus aureus*. The synergistic effects decrease with an increasing number of double bonds in the chain. A possible explanation for this effect is that the introduction of unsaturation or branching into the hydrophobic groups increases the surfactant water solubility and, therefore, increases the activity [44]. The high toxicity of iCNSL at the highest test concentration in this bioassay was most likely due to the presence of anacardic acid and to its additive and/or synergistic effects.

Cardanol may account for the toxic activity of tCNSL in the* A. salina* bioassay (Figure 3). Studies of the wastewater from cashew-processing factories show that cardanol, which is the main component of tCNSL, is toxic to* A. salina* [45]. The iCNSL contains primarily anacardic acids, and tCNSL contains primarily cardanols. This difference confounds conclusions regarding whether anacardic acids or cardanol was the active agent responsible for the effects observed in this study.

Toxicity was also characterised in this study by a reduction in the growth of* A. cepa* root meristems. The highest concentration of iCNSL (69.50 *μ*g·mL^−1^) caused significant root growth inhibition (*P* < 0.001) compared with the negative control. However, toxic activity was not observed for any of the three concentrations of tCNSL (Figure 3).

The iCNSL concentration of 69.50 *μ*g·mL^−1^ was more than double the IC_50_ that was observed in* A. salina* (36.96 *μ*g·mL^−1^), but the two lower concentrations were not toxic to* A. cepa *root meristems (Figure 3). Another study, in which three species of molluscs of the same genus (*Biomphalaria straminea, B. tenagophila, *and* B. glabrata*) were treated with a 20 ppm hexane extract, showed mortality rates that ranged from 97.1% to 100% after 24 hours of exposure [46].

The* A. cepa* test system is a key* in vivo* model for the evaluation of root growth after direct treatment with a substance of interest and for the prediction of DNA damage. The test is considered to be an effective preselection tool for toxicity and genotoxicity studies [47] because the results can be extrapolated to other animals and plants [47].

The* A. cepa* test also provides other macroscopic parameters that indicate the toxicity of chemicals and environmental pollutants. These toxicity parameters include very large roots, which indicate cellular proliferation; colour changes; and the presence of tumours [48]. This test reveals toxic [26] and cytotoxic effects [49].

The effects of extracts against damage caused by toxic agents have been analysed in recent studies assessing root size via the* A. cepa* test [28, 50]. Certain metals, including copper, may inhibit root growth, most likely by inhibiting cell division, and may also cause toxicity and cytotoxicity [51].

In the present study, only the highest test concentration of tCNSL showed preventive effects when exposed to the CuSO_4_·5H_2_O solution in* A. cepa* meristems. However, neither iCNSL nor tCNSL prevented the toxicity induced by CuSO_4_·H_2_O in co- or posttreatment applications (Table 3).

Root growth is regulated by the combination of cell division activity in mitotically active meristems and cell elongation in the regions that are proximal to root apices [52]. Only the highest concentration of iCNSL had significant antiproliferative activity (*P* < 0.05) compared with the negative control (Figure 5). This finding suggests that this concentration caused disturbance in meristem proliferation in* A. cepa*.

Macroscopic parameters are associated with toxicity and may likewise be associated with a reduction in the MI, which would affect DNA replication and protein synthesis [53]. No preventive, modulating, and reparative activities of CNSL against the cytotoxicity induced by copper sulphate were observed in this study (Table 4).

Oliveira et al. [23] also found that anacardic acid is a larger component of iCNSL than of tCNSL (Table 1) and that anacardic acid may have had prooxidant effects in* A. cepa* meristems. Recent studies indicate that antiproliferative effects on mammalian cell cultures are associated with oxidative stress [54] because the production of reactive oxygen species (ROS) impacts root growth and may inhibit growth and cell division [55].

The tCNSL showed no cytotoxic effect (*P* > 0.05) at any concentration. A positive correlation was found between inhibited root growth and reduced MI at the highest iCNSL concentration (Figures 4 and 5).

The MI is calculated by dividing the number of dividing cells by the total number of cells observed and is expressed as a percentage [56]. A reduction in the MI can be interpreted as cell death [57]. The present study showed a mitodepressive effect of iCNSL at the highest test concentration (69.50 *μ*g·mL^−1^) on* A. cepa* cell division. The mitodepressive effect may have resulted from abnormal cellular conditions caused by the treatment. The reduction in the MI may have been related to early prophase arrest [58], inhibition of DNA synthesis, or cell cycle arrest at the G2 phase, which would prevent cells from entering mitosis [59]. The reduced MI also inhibits microtubule formation and nucleoprotein synthesis and reduces the ATP levels that provide energy for spindle elongation, microtubule dynamics, and chromosome movement [60].

Kubo et al. [61] also showed that anacardic acid and cardol may have a moderate cytotoxic effect. The inhibition of prooxidant enzymes may account for this effect. The volume of the hydrophobic side chain and its ability to act as a surfactant would explain its cytotoxic effect. Cardol has also been shown to be cytotoxic at a dose of 0.01 mM in HeLa cells [62]. The results of these two studies might confirm the cytotoxic action of the highest concentration of iCNSL which contains anacardic acid and cardol [18].

Acevedo et al. [22] showed that the anacardic acid present in* Amphipterygium adstringens* has cytotoxic effects in the peripheral lymphocytes of mice treated with doses of less than 10 mg/kg. The cytotoxic effects were evidenced by decreases in polychromatic and normochromatic erythrocytes. Anacardic acid from* A. adstringens *is also cytotoxic against Gram-positive bacteria in dental abscesses, has molluscicidal activity [63], inhibits apoptosis in chick embryonic neuronal cells [64], and inhibits breast cancer (MCF-7 and MDA-MB 231) cervical cancer cell lines and other types of tissues, including lung, liver, bladder, and melanoma [65].

Recent studies have also shown that a combination of anacardic acid and lunasin, which is another natural plant extract, may exhibit anticarcinogenic properties. These compounds act on the regulation of the expression of several genes involved in the cell cycle, apoptosis, and signal transduction [66]. Both compounds have a strong inhibitory effect on a number of cancer cell lines [67–69]. For example, [70] reported inhibition of the growth of HepG2 and U266 tumour cells treated with 60 *µ*M of anacardic acid for 24, 48, and 72 hours.

The frequencies of CA and MN are commonly used to detect genotoxicity [59, 71]. This study investigated genotoxicity based on the frequency of MN and CAs, such as chromosome bridges, vagrant and laggard chromosomes, and chromosome fragments (Figure 7).

Iarmarcovai et al. [72] characterise micronuclei as small, spherical bodies that consist of genetic material that is not incorporated into the main nucleus during the final stages of mitosis. MN may result from the failure of acentric chromosome fragments to incorporate into the cell nucleus and clastogenicity (DNA breaks) or from whole chromosomes of aneugenic origin (disturbance in the mitotic spindle). The iCNSL at a concentration of 69.50 *µ*g·mL^−1^ is thought to have induced genotoxic effects by means of clastogenic mechanisms (Table 5) because the MN that were generated at this concentration are considered small. Small MN are indicative of clastogenic action [73] resulting from genotoxic stress [74, 75].

The mutagenic response that occurred at the highest iCNSL concentration might have resulted from chromosomal instability, phenotypes, and cellular changes caused by genetic defects and/or exogenous exposure [76]. However, previous studies found that iCNSL did not have a mutagenic effect in the Ames test [22] or the MN test in mice bone marrow [77].

We, therefore, hypothesise that the MN formed at the highest iCNSL concentration resulted from breaks that occurred during cell division, possibly due to unrepaired or incorrectly repaired damage or to poor chromosome separation as result of mitotic malfunction. These events may have resulted from oxidative stress [78] and therefore from an integrated response to instability of the genetic material [79] that reflected various chromosomal changes [71].

However, iCNSL and tCNSL had no significant genotoxic effects on the frequency of CAs compared with the negative control (Figure 7). The iCNSL at the highest test concentration showed no preventive, antimutagenic, and reparative responses against CuSO_4_·5H_2_O. The iCNSL at the two lower concentrations and tCNSL did show preventive, antimutagenic, and reparative activities, as indicated by the reduced frequency of MN (Table 5). These results are consistent with the decrease in the frequency of CAs that resulted from the inhibition of damage induced by CuSO_4_·5H_2_O (Table 5).

Several experimental models have shown that synthetic or natural resorcinolic lipids do not cause DNA damage at low concentrations, which suggests that they have anticancer activity [80]. The results of these studies are consistent with the results of the present study, in which no genotoxicity was found at the lowest concentrations of iCNSL and tCNSL.

The present study documented the genotoxic effects of CuSO_4_·5H_2_O, which are explained by the ability of it causing DNA damage [29]. The chemical components of iCNSL and tCNSL may have protected, modulated, and repaired the oxidative effects of CuSO_4_·5H_2_O in* A. cepa* meristems. Cardol and cardanol were found to exhibit* in vitro* antioxidant effects in studies of the chemical characteristics of CNSL. These compounds have these effects because they scavenge free radicals, including the hydroxyl radical [23].

Other studies have also reported that the genotoxicity of chemical agents may be repaired by phenolic compounds with antioxidant and radical-scavenging activities [81]. Chromosomal aberrations consist of changes in chromosome structure that result in breaks or exchange of chromosomal material. These types of damage are usually lethal to cells, but some are viable and may have somatic or hereditary genetic effects [82].

Chromosomal fragments in cells indicate chromosomal breaks and may be related to anaphase bridges [83], disturbances in microtubule assembly, and cell death [84]. The results of the present study (Table 5) show that iCNSL and tCNSL failed to induce the fragment type of CA and therefore did not cause anaphase bridges when compared to the control group.

Antimutagenic compounds are able to induce some metabolic enzymes that may act as enzymatic inhibitors of mutagenic agents or inhibitors of promutagens in pretreatment experiments [85, 86]. The preventive, antimutagenic, and reparative effects of iCNSL and tCNSL observed in* A. cepa* (Tables 4 and 5) (except at the highest iCNSL concentration) were corroborated with the study of [15], who reported that tCNSL effect protected against oxidative stress (at a concentration of 100–500 *μ*g·mL^−1^) in* S. cerevisiae* that were defective in antioxidant enzymes. The protection against damage caused by H_2_O_2_ occurs via bioantimutagenic mechanisms, but it occurs by means of dysmutagenesis in concurrent treatment. Bioantimutagenic agents act on the physiological mechanisms of DNA protection and repair and reverse the mutagenic effects and prevent their persistence [85, 87]. Thus, CNSL most likely acted as a bioantimutagenic and dysmutagenic agent and showed a stronger antimutagenic effect.

Components of phenolic lipids, including anacardic acid and alkylresorcinol, have antigenotoxic activity* in vitro* because of the ability of lipids to interact with biological membranes [88]. This is confirmed by the presence of hydrophilic and hydrophobic regions in their structures, which give lipids an amphipathic character that is responsible for their affinity for biological membranes. This character allows the phenolic lipids to be incorporated easily into cell membranes [89].

Cardanol also has antioxidant effects [23], and phenolic compounds with this ability can suppress genotoxicity [81]. Deszcz and Kozubek [90] noted that alkylresorcinols may be characterised as antioxidants when they are at very low concentrations, and they protect free fatty acids and phospholipids against peroxidation induced by the iron and autooxidation of biological membranes. These activities may constitute the main factor accounting for the antimutagenic activity exhibited by these compounds.

The present study confirms the strong antimutagenic, preventive, and restorative effects of CNSL. De Lima et al. [25] observed that iCNSL (at a concentration of 200 *μ*g·mL^−1^) had an antioxidant effect in* S. cerevisiae*. Andrade et al. [15] observed that 100 *μ*g·mL^−1^ of tCNSL might reduce free radical levels by 88.9% in the DPPH test and that it scavenges hydroxyl radicals by means of xanthine oxidase, resulting in antioxidant activity with an IC_50_ = 702 *µ*g/mL.

Melo Cavalcante et al. [91] also confirmed that* A. occidentale* pulp has antioxidant effects against H_2_O_2_ at pre-, co-, and posttreatment in* Salmonella typhimurium*, as assessed by the Ames test. The authors attributed these effects to the pulp's chemical components, which include anacardic acid. These components may also protect* S. typhimurium* (TA102) against the damage induced by aflatoxin B_1_ via several mechanisms [92]. Cashew juice and cajuina (processed juice) reduce damage to the peripheral blood cells of mice. The juice caused a 60.82% reduction in damage and the cajuina caused an 82.19% reduction in damage, compared with cyclophosphamide. Further, the juice and the cajuina reduced the number of CAs in the bone marrow of mice by 53% and 65%, respectively. These effects may be related to the antioxidant activities of their components [13]. The results reported by de Carvalho Melo-Cavalcante et al. [13] confirm the (concentration-dependent) antimutagenic and antigenotoxic effects of iCNSL and tCNSL observed in the present study.

## 5. Conclusions

In summary, this study showed that anacardic acids are the primary components of iCNSL and cardanol of tCNSL. Both iCNSL and tCNSL showed protective (pretreatment), modulating (cotreatment), and reparative (posttreatment)* in vivo* effects against the damage induced by copper sulphate in* A. cepa* meristems at the lowest concentrations evaluated. Therefore, CNSL, which is a natural and renewable product extracted from the cashew nut shell, can be the basis for further studies to determine the mechanisms activated by its components and the mechanisms by which their synergism produces beneficial effects. These studies might be precursors for the production of biotechnological products.

## Figures and Tables

**Table 1:** The investigated components of iCNSL and tCNSL.

Natural iCNSL^*^			
Peak	Retention time (min)	Compounds	Yield
1	36.80	Metilcardol	2.90
2	37.60	Monounsaturated anacardic acid	82.90
3	37.62	Diunsaturated anacardic acid	8.00
4	37.70	Anacardic acid	3.60
5	38.05	Unidentified	2.60

Technical tCNSL			
Peak	Retention time (min)	Compounds	Yield

6	18.08	Monounsaturated cardanol	79.40
7	18.10	Diunsaturated cardanol	8.67
8	18.17	Cardanol	3.23
9	20.71	Unidentified	8.70

^*^Analysed in the form of methyl esters.

**Table 2:** Antitoxicity of iCNSL and tCNSL as measured by the effect on  *A. cepa* root growth (cm; mean ± SD, and % growth inhibition) at different concentrations and treatments with copper sulphate (1.2 *μ*g·mL^−1^).

Treatments (% inhibition)	Controls/vehicle			iCNSL			tCNSL		
	NC	PC	TWEEN	Concentrations			Concentrations		
				17.37 *μ*g·mL^−1^	34.75 *μ*g·mL^−1^	69.50 *μ*g·mL^−1^	17.37 *μ*g·mL^−1^	34.75 *μ*g·mL^−1^	69.50 *μ*g·mL^−1^
Pretreatment (% inhibition)	NT	NT	NT	0.51 ± 0.17^a∗∗∗,b∗∗^ (62.78%)	0.55 ± 0.18^a∗∗∗,b∗∗^ (59.86%)	0.55 ± 0.12^a∗∗∗,b∗∗^ (59.86%)	0.45 ± 0.23^a∗∗∗.b∗^ (67.16%)	0.71 ± 0.26^a∗∗∗,b∗∗∗^ (48.18%)	1.34 ± 0.25^b∗∗∗^ (2.19%)
Cotreatment (% inhibition)	NT	NT	NT	0.23 ± 0.09^a∗∗∗^ (83.09%)	0.34 ± 0.27^a∗∗∗^ (75.00%)	0.38 ± 0.24^a∗∗∗^ (72.03%)	0.46 ± 0.09^a∗∗∗;b∗^ (66.18%)	0.48 ± 0.20^a∗∗∗;b∗^ (64.71%)	0.52 ± 0.32^a∗∗∗;b∗∗^ (61.23%)
Posttreatment (% inhibition)	NT	NT	NT	0.32 ± 0.09^a∗∗∗^ (83.09%)	0.33 ± 0.11^a∗∗∗^ (75.00%)	0.40 ± 0.19^a∗∗∗;b∗^ (72.00%)	0.36 ± 0.07^a∗∗∗^ (66.18%)	0.38 ± 0.09^a∗∗∗^ (64.71%)	0.32 ± 0.05^a∗∗∗^ (61.77%)

NC (dechlorinated water). PC (copper sulphate solution). TWEEN (solvent). NT (not tested). 1.000 cells evaluated per bulb, totalling 5,000 cells per group.  ^a^Significant compared with the negative control; ^b^significant compared with the positive control. ^*^ *P* < 0.05, ^**^ *P* < 0.01, and ^***^ *P* < 0.001 ANOVA. Tukey's test for multiple comparisons between groups.

**Table 3:** Anticytotoxicity of iCNSL and tCNSL as measured by the effect on the cell division rate of *A. cepa* roots (cm; mean ± standard deviation, and % cell division inhibition) at different concentrations and treatments with copper sulphate (1.2 *μ*g·mL^−1^).

Treatments (% inhibition)	Controls/vehicle			iCNSL			tCNSL		
	NC	PC	TWEEN	Concentrations			Concentrations		
				17.37 *µ*g·mL^−1^	34.75 *µ*g·mL^−1^	69.50 *µ*g·mL^−1^	17.37 *µ*g·mL^−1^	34.75 *µ*g·mL^−1^	69.50 *µ*g·mL^−1^
Pretreatment (% inhibition)	NT	NT	NT	29.28 ± 6.37^a∗∗∗;b∗∗^ (44.54%)	23.78 ± 0.68^a∗∗∗^ (54.96%)	28.14 ± 1.33^a∗∗∗;b∗∗^ (46.70%)	8.92 ± 0.75^a∗∗∗^ (83.10%)	9.68 ± 1.46^a∗∗∗^ (81.66%)	13.54 ± 3.44^a∗∗∗^ (74.35%)
Cotreatment (% inhibition)	NT	NT	NT	24.48 ± 3.89^a∗∗∗;b∗^ (53.63%)	23.16 ± 1.91^a∗∗∗^ (56.13%)	27.28 ± 2.16^a∗∗∗;b∗∗ ^ (48.33%)	8.38 ± 0.84^a∗∗∗^ (84.12%)	9.12 ± 1.46^a∗∗∗^ (82.72%)	14.84 ± 2.76^a∗∗∗^ (71.89%)
Posttreatment (% inhibition)	NT	NT	NT	25.64 ± 1.86^a∗∗∗^ (51.43%)	20.64 ± 2.85^a∗∗∗^ (60.90%)	28.52 ± 1.58^a∗∗∗;b∗∗∗^ (45.98%)	13.16 ± 1.23^a∗∗∗^ (75.07%)	21.72 ± 2.08^a∗∗∗^ (58.86%)	32.00 ± 1.30^a∗∗∗;b∗∗∗^ (39.39%)

NC (dechlorinated water). PC (copper sulphate solution). TWEEN (solvent). NT (not tested). 1.000 cells evaluated per bulb, totalling 5,000 cells per group. ^a^Significant compared with the negative control; ^b^significant compared with the positive control. ^*^ *P* < 0.05, ^**^ *P* < 0.01, and ^***^ *P* < 0.001 ANOVA. Tukey's test for multiple comparisons between groups.

**Table 4:** Mutagenicity and antimutagenicity of iCNSL and tCNSL as measured by the number of micronuclei (mean ± SD of 1.000 cells per slide, 5 slides per test group) in *A. cepa* root meristems at different concentrations and treatments with copper sulphate (1.2 *µ*g·mL^−1^).

Treatments	Controls/vehicle			iCNSL			tCNSL		
	NC	PC	TWEEN	Concentrations			Concentrations		
				17.37 *µ*g·mL^−1^	34.75 *µ*g·mL^−1^	69.50 *µ*g·mL^−1^	17.37 *µ*g·mL^−1^	34.75 *µ*g·mL^−1^	69.50 *µ*g·mL^−1^
Without treatment	1.20 ± 1.09	8.40 ± 1.14^a∗∗^	11.80 ± 0.83	1.80 ± 0.83	1.80 ± 1.48	4.00 ± 0.70^∗a^	1.00 ± 1.22	1.20 ± 0.83	2.20 ± 0.83
Pretreatment	NT	NT	NT	1.40 ± 1.34^b∗∗∗^	1.00 ± 0.90^b∗∗∗^	4.20 ± 1.30^a∗;b∗∗∗^	2.20 ± 2.16^b∗∗∗^	1.80 ± 0.90^b∗∗∗^	2.00 ± 1.41^b∗∗∗^
Cotreatment	NT	NT	NT	1.60 ± 0.89^b∗^	1.80 ± 1.48^b∗^	5.40 ± 1.67^a∗∗;b∗∗^	1.00 ± 1.00^b∗∗∗^	1.00 ± 0.70^b∗∗∗^	2.00 ± 1.58^b∗∗∗^
Posttreatment	NT	NT	NT	2.2 ± 148^b∗∗∗^	1.00 ± 1.22^b∗∗∗^	4.20 ± 1.48^a∗;b∗∗∗^	1.40 ± 1.14^b∗∗∗^	1.60 ± 1.51^b∗∗∗^	1.80 ± 0.83^b∗∗∗^

NC (dechlorinated water). PC (copper sulphate solution). TWEEN (solvent). NT (not tested). 5.000 cells evaluated per bulb, totalling 5.000 per group. ^a^Significant compared with the negative control; ^b^significant compared with the positive control. ^*^ *P* < 0.05, ^**^ *P* < 0.01, and ^***^ *P* < 0.001 ANOVA. Tukey's test for multiple comparisons between groups.

**Table 5:** Antimutagenicity of iCNSL and tCNSL as measured by the frequency of chromosomal aberrations (mean ± SD of 1.000 cells per slide, 5 slides per test group) in *A. cepa* root meristems at different concentrations (pre-, co-, and posttreatment) with copper sulphate (1.2 *µ*g·mL^−1^).

Groups with concentration	Chromosome bridges	Chromosomal aberration/5000 cells				Total frequency (%)
		Vagrant chromosomes	Laggard chromosomes	Fragments	Total frequency	
iCNSL pretreatment						
17,37 *µ*g·mL^−1^	0.40 ± 0.89^b∗^	1.60 ± 0.50^b∗∗∗^	4.00 ± 4.84	2.00 ± 1.22	8.00 ± 3.46^b∗∗∗^	0.80 ± 0.34^b∗∗∗^
34,75 *µ*g·mL^−1^	0.60 ± 0.89^b∗^	1.20 ± 1.09^b∗∗∗^	2.60 ± 2.96^b∗^	2.60 ± 1.94	7.00 ± 4.06^b∗∗∗^	0.70 ± 0.40^b∗∗∗^
tCNSL pretreatment						
17,37 *µ*g·mL^−1^	1.20 ± 1.00	1.00 ± 0.00^b∗∗∗^	8.00 ± 4.41	1.20 ± 0.83^b∗^	11.40 ± 7.33^b∗∗∗^	1.14 ± 0.73^b∗∗∗^
34,75 *µ*g·mL^−1^	0.75 ± 0.50	1.20 ± 0.44^b∗∗∗^	8.20 ± 3.11	2.40 ± 1.51	12.80 ± 3.76^b∗∗∗^	1.28 ± 0.37^b∗∗∗^
69,5 *µ*g·mL^−1^	0.80 ± 0.40	1.60 ± 0.44^b∗∗∗^	8.00 ± 4.00	2.60 ± 1.81	13.00 ± 5.61^b∗∗∗^	1.30 ± 0.56^b∗∗∗^
iCNSL cotreatment						
17,37 *µ*g·mL^−1^	0.40 ± 0.89^b∗^	1.00 ± 0.00^b∗∗∗^	3.40 ± 1.81^b∗^	1.60 ± 1.14	6.40 ± 2.51^b∗∗∗^	0.64 ± 0.25^b∗∗∗^
34,75 *µ*g·mL^−1^	0.20 ± 0.44^b∗^	1.20 ± 0.44^b∗∗∗^	5.40 ± 2.60	1.00 ± 0.70^b∗∗^	7.80 ± 2.77^b∗∗∗^	0.78 ± 0.27^b∗∗∗^
tCNSL cotreatment						
17,37 *µ*g·mL^−1^	0.60 ± 0.54^b∗^	1.60 ± 1.14^b∗∗∗^	5.20 ± 1.78	2.00 ± 1.00	9.40 ± 2.51^b∗∗∗^	0.94 ± 0.25^b∗∗∗^
34,75 *µ*g·mL^−1^	0.60 ± 0.54^b∗^	1.20 ± 1.64^b∗∗∗^	4.60 ± 2.19	2.40 ± 1.14	8.80 ± 0.30^b∗∗∗^	8.80 ± 3.03^b∗∗∗^
69,50 *µ*g·mL^−1^	1.60 ± 1.94	3.40 ± 1.67^b∗∗∗^	8.80 ± 5.35	3.40 ± 2.79	17.20 ± 5.76^b∗∗∗^	1.72 ± 0.57^b∗∗∗^
iCNSL posttreatment						
17,37 *µ*g·mL^−1^	0.20 ± 0.44^b∗∗^	1.20 ± 0.83^b∗∗∗^	1.60 ± 1.57^b∗∗^	1.60 ± 1.14	4.60 ± 1.81^b∗∗∗^	0.46 ± 0.18^b∗∗∗^
34,75 *µ*g·mL^−1^	0.20 ± 0,44^b∗^	2.00 ± 1.00^b∗∗∗^	4.00 ± 4.35	2.40 ± 0.89	8.60 ± 4.27^b∗∗∗^	0.86 ± 0.42^b∗∗∗^
tCNSL posttreatment						
17,37 *µ*g·mL^−1^	1.20 ± 0.44	1.00 ± 1.22^b∗∗∗^	6.00 ± 4.84	1.00 ± 1.22^b∗^	9.20 ± 7.46^b∗∗∗^	0.92 ± 0.74^b∗∗∗^
34,75 *µ*g·mL^−1^	0.80 ± 0.44	2.60 ± 1.51^b∗∗∗^	7.40 ± 2.52	2.60 ± 2.60	13.00 ± 6.51^b∗∗∗^	1.30 ± 0.65^b∗∗∗^
69,50 *µ*g·mL^−1^	1.20 ± 0.44	3.80 ± 1.78^b∗∗^	7.00 ± 4.12	2.80 ± 0.83	14.80 ± 4.08^b∗∗^	1.48 ± 0.40^b∗∗^

NC (dechlorinated water). PC (copper sulphate solution). TWEEN (solvent). NT (not tested). 5.000 cells evaluated per bulb, totalling 5,000 per group. ^a^Significant compared with the negative control; ^b^significant compared with the positive control. ^*^ *P* < 0.05, ^**^ *P* < 0.01, and ^***^ *P* < 0.001 ANOVA. Tukey's test for multiple comparisons between groups.
